# Case-only analysis of routine surveillance data: detection of increased vaccine breakthrough infections with SARS-CoV-2 variants in Europe

**DOI:** 10.1017/S0950268824001833

**Published:** 2025-01-06

**Authors:** Jeremy Brown, Piers Mook, Maarten Vanhaverbeke, Amy Gimma, José Hagan, Isaac Singini, Mária Avdičová, Gillian Cullen, Liidia Dotsenko, Joël Mossong, Malgorzata Sadkowska-Todys, Heelene Suija, Nick Bundle, Richard Pebody

**Affiliations:** 1 World Health Organization (WHO) Regional Office for Europe, Copenhagen, Denmark; 2 The Regional Authority of Public Health in Banska Bystrica, Public Health Authority of the Slovak Republic; 3 Health Protection Surveillance Centre, Dublin, Republic of Ireland; 4 Health Board, Tallinn, Republic of Estonia; 5 Health Directorate, Luxembourg, Luxembourg; 6 National Institute of Public Health NIH – National Research Institute, Warsaw, Poland; 7 European Centre for Disease Prevention and Control (ECDC), Stockholm, Sweden

## Abstract

With the ongoing emergence of SARS-CoV-2 variants, there is a need for standard approaches to characterize the risk of vaccine breakthrough. We aimed to estimate the association between variant and vaccination status in case-only surveillance data. Included cases were symptomatic adult laboratory-confirmed COVID-19 cases, with onset between January 2021 and April 2022, reported by five European countries (Estonia, Ireland, Luxembourg, Poland, and Slovakia) to The European Surveillance System. Associations between variant and vaccination status were estimated using conditional logistic regression, within strata of country and calendar date, and adjusting for age and sex. We included 80,143 cases including 20,244 Alpha (B.1.1.7), 152 Beta (B.1.351), 39,900 Delta (B.1.617.2), 361 Gamma (P.1), 10,014 Omicron BA.1, and 9,472 Omicron BA.2. Partially vaccinated cases were more likely than unvaccinated cases to be Beta than Alpha (adjusted odds ratio [aOR] 2.48, 95% CI 1.29–4.74), and Delta than Alpha (aOR 1.75, 1.31–2.34). Fully vaccinated cases were relative to unvaccinated cases more frequently Beta than Alpha (aOR 4.61, 1.89–11.21), Delta than Alpha (aOR 2.30, 1.55–3.39), and Omicron BA.1 than Delta (aOR 1.91, 1.60–2.28). We found signals of increased breakthrough infections for Delta and Beta relative to Alpha, and Omicron BA.1 relative to Delta.

## Introduction

Vaccination is a key strategy for the reduction in transmission, morbidity, and mortality of infectious diseases. The efficacy of licensed COVID-19 vaccines, as estimated in randomized controlled trials, is high.[1,2] However, some real-world effectiveness estimates are lower and there is evidence that the effectiveness of currently licensed COVID-19 vaccines against infection may be lower against more recent circulating SARS-CoV-2 variants of concern (VOC). Case-only analytical approaches have been identified to have the potential for the rapid evaluation of the interaction between SARS-CoV-2 variants and COVID-19 vaccine effectiveness.[3,4] We aimed to estimate the odds ratio between vaccination status and SARS-CoV-2 variants among cases using routine surveillance data to identify signals of increased vaccine breakthrough with specific variants.

## Methods

### Study population

We identified symptomatic COVID-19 laboratory-confirmed cases with complete data on age, sex, vaccination status, date of onset, and vaccination date submitted to The European Surveillance System (TESSy) database as part of regional COVID-19 surveillance, which is jointly coordinated by the WHO Regional Office for Europe and the European Centre for Disease Prevention and Control (ECDC). These data were submitted by five EU Member States (Estonia, Ireland, Luxembourg, Poland, and Slovakia).

We selected adult cases (≥18 years of age) with the date of onset between 1st January 2021 and either 19th April 2022 (Estonia, Luxembourg, and Slovakia) or 12th December 2021 (Ireland and Poland) with one of the following SARS-CoV-2 variants: Alpha (B.1.1.7), Beta (B.1.351), Delta (B.1.617.2), Gamma (P.1), Omicron BA.1, and Omicron BA.2. Sublineages of VOCs (e.g., BA.2 + L452X) were categorized with their parent lineage (e.g., BA.2).

Cases from Ireland and Poland were restricted to those with onset before 13th December 2021 given later changes to reporting by these countries. Similarly, the few cases that received two booster doses were excluded, as there were insufficient numbers of these cases to allow comparison with the unvaccinated.

### Study design

If vaccination is equally effective against two different VOCs then we anticipate, for a given location and time, the relative frequency of these two variants among unvaccinated and vaccinated cases will be the same. However, if vaccination is less effective against one VOC, then a higher proportion of infections among the vaccinated will be for that VOC relative to infections among the unvaccinated.

The odds ratio for VOC relative to reference variant among COVID-19 cases was estimated stratified by date of onset and report country. Under certain assumptions, the estimated odds ratio in a case-only analysis is equivalent to the relative risk of infection by vaccination status (i.e. one minus vaccine effectiveness) for VOC divided by the relative risk of infection by vaccination status for reference variant (equation 1).[5–9] The use of case-only data to estimate a ratio of relative risks has been commonly used to estimate gene–gene and gene–environment interactions[6,7,10], but can also be used to estimate a ratio of relative risks between variant and vaccine effectiveness, in what is known as a sieve analysis[8,9,11], under the assumption of independence of vaccination status and variant exposure. Sieve analysis has typically been applied to randomized trials, where independence of vaccination status and variant exposure is expected, and there has been limited application of this approach in observational data or the surveillance setting. In the observational setting, independence of variant exposure and vaccination status is unlikely given differences in risk-related behaviour by vaccine status. However, an assumption that the relative frequency of exposure to different variants is the same in vaccinated and unvaccinated is reasonable for community transmission at a given date and location.
(1)

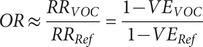

There is a close similarity between this approach and the test-negative design where the distribution of vaccination in cases is compared with non-cases who also present for testing rather than between cases of different variants.[12,13] In the test-negative design, we can assume there is no vaccine efficacy against other infectious agents causing presentation (e.g., different viruses) and the denominator of equation 1 can be assumed to be one, allowing direct estimation of vaccine efficacy. This similarity is apparent in what is often considered the earliest test-negative design [12], in which the distribution of pneumococcal serotypes was compared in cases of pneumococcal infection with and without prior pneumococcal vaccination under the presumption of no vaccine efficacy against serotypes not included in the vaccine.[14] As in test-negative designs, confounding bias by healthcare-seeking behaviour is potentially reduced by restriction to a population who presents to healthcare if infected.[12]

### Outcome

We estimated the odds for VOC relative to reference variant for variants that co-circulated together, comparing Beta to Alpha [ref], Delta to Alpha [ref], Gamma to Alpha [ref], Omicron BA.1 to Delta [ref], and Omicron BA.2 to Omicron BA.1 [ref]. For each comparison, we restricted analysis to cases with either VOC or reference variant and to days for each country in which cases of both variants were reported.

### Exposure

The exposure variable of interest was COVID-19 vaccination status. Unvaccinated cases were defined as cases with no vaccination date or with vaccination after the date of symptom onset. Partially vaccinated cases were defined as cases with a date of onset >14 days after the date of first dose (excluding single-dose vaccines, i.e., Janssen Ad26.COV2-S) and with no second dose. Fully vaccinated cases were defined as cases with a date of onset >14 days after the second dose (or first dose for single-dose vaccines) and with no additional dose. Additionally, vaccinated cases were defined as cases with a date of onset >14 days after the third dose (or second dose for single-dose vaccines) and with no further dose.

### Covariates

We adjusted for country and date, as well as age and sex. Age was categorized into the following groups: 15–24, 25–49, 50–64, 65–79, and 80+ years.

### Statistical analysis

Descriptive statistics, stratified by vaccination status, were calculated for included cases.

For the primary analysis, for each comparison of two SARS-CoV-2 variants, odds ratios were estimated using conditional logistic regression conditional on strata of country and calendar date (by day) and adjusting for age and sex. As a secondary analysis, the association between the SARS-CoV-2 variant and vaccination status was assessed by specific vaccine (e.g., Ad26.COV2-S – Janssen). For this analysis, vaccinated cases were restricted to those receiving the most common vaccines in the included countries Ad26.COV2-S (Janssen), BNT162b2 (Pfizer/BioNTech), and ChAdOx1 nCoV-19 (AstraZeneca) and to comparisons where there were >30 cases in each exposure group to avoid sparse data bias in odds ratio estimation using conditional logistic regression.[15] A further secondary analysis examined whether the association between the variant and full vaccination differed by time since vaccination (categorized <3 or ≥3 months) with the 3-month cut-off chosen given evidence of decreasing vaccine effectiveness after 100 days following full vaccination.[16]

Wald tests were used to test the associations between vaccination status and the SARS-CoV-2 variant. Likelihood ratio tests were used to test whether the vaccination status-variant association differed by vaccine and time since vaccination.

### Sensitivity analysis

An association between vaccination status and variant may arise among those exposed to COVID-19, due to travellers, who may be highly vaccinated due to travel restrictions, importing in a new variant. This will be particularly problematic in the early stages of variant transmission in a country. As a result, travel history may be a common cause of vaccination status and SARS-CoV-2 variant exposure. To assess potential bias due to this, a sensitivity analysis was conducted whereby cases were excluded if they were imported or had missing import status.

Data analyses were conducted using R (4.0.3).

## Results

We selected for inclusion 80,143 adult symptomatic cases (see Appendix Figure 1 for the study flow chart). More cases were Alpha (20,244, 25.3%), Delta (39,900, 49.8%), Omicron BA.1 (10,014, 12.5%), or Omicron BA.2 (9,472, 11.8%) than Beta (152, 0.2%) or Gamma (361, 0.5%) (see Table 1). Among vaccinated cases with recorded vaccine names, the most common vaccine administered at first dose was BNT162b2 (Pfizer/BioNTech; 18,697 of 29,202, 64.0%).

**Figure 1.**
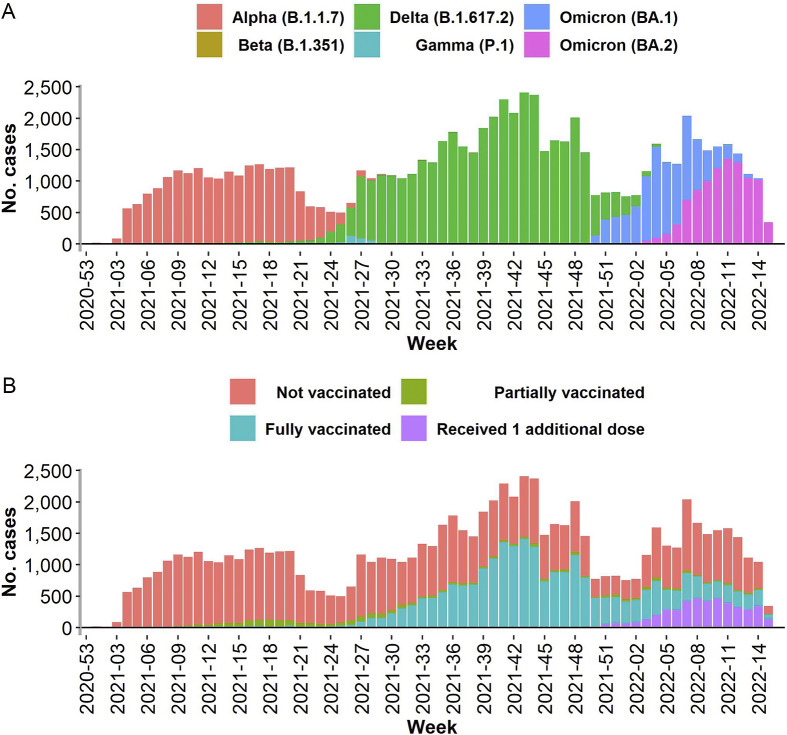
Weekly count of included cases (a) by variant and (b) by vaccination status. *Note:* Univariable and multivariable conditional logistic regression models were fitted within strata of report country and date.

**Table 1.** Characteristics of included cases by vaccination status

Characteristic	Not vaccinated, N = 49,935	Partially vaccinated, N = 2,521	Fully vaccinated, N = 23,088	Received 1 additional dose, N = 4,599	Overall, N = 80,143
**Virus variant**					
Alpha (B.1.1.7)	19,011 (38.1)	941 (37.3)	278 (1.2)	14 (0.3)	20,244 (25.3)
Beta (B.1.351)	121 (0.2)	20 (0.8)	11 (0.0)	0 (0.0)	152 (0.2)
Delta (B.1.617.2)	21,145 (42.3)	1,098 (43.6)	17,558 (76.0)	99 (2.2)	39,900 (49.8)
Gamma (P.1)	255 (0.5)	61 (2.4)	45 (0.2)	0 (0.0)	361 (0.5)
Omicron (BA.1)	4,727 (9.5)	229 (9.1)	3,231 (14.0)	1,827 (39.7)	10,014 (12.5)
Omicron (BA.2)	4,676 (9.4)	172 (6.8)	1,965 (8.5)	2,659 (57.8)	9,472 (11.8)
**Country or area**					
Estonia	1,873 (3.8)	120 (4.8)	924 (4.0)	84 (1.8)	3,001 (3.7)
Ireland	11,042 (22.1)	582 (23.1)	4,366 (18.9)	0 (0.0)	15,990 (20.0)
Luxembourg	4,227 (8.5)	447 (17.7)	5,577 (24.2)	4,454 (96.8)	14,705 (18.3)
Poland	11,074 (22.2)	598 (23.7)	4,933 (21.4)	61 (1.3)	16,666 (20.8)
Slovakia	21,719 (43.5)	774 (30.7)	7,288 (31.6)	0 (0.0)	29,781 (37.2)
**Hospitalized**					
Yes	3,277 (7.9)	146 (6.6)	809 (4.0)	86 (1.9)	4,318 (6.3)
No	38,407 (92.1)	2,067 (93.4)	19,587 (96.0)	4,492 (98.1)	64,553 (93.7)
Missing	8,251	308	2,692	21	11,272
**Imported**					
Yes	756 (1.8)	62 (3.6)	561 (3.8)	52 (42.3)	1,431 (2.4)
No	41,031 (98.2)	1,680 (96.4)	14,295 (96.2)	71 (57.7)	57,077 (97.6)
Missing	8,148	779	8,232	4,476	21,635
**Sex**					
Female	26,925 (53.9)	1,411 (56.0)	13,044 (56.5)	2,564 (55.8)	43,944 (54.8)
Male	23,010 (46.1)	1,110 (44.0)	10,044 (43.5)	2,035 (44.2)	36,199 (45.2)
**Age**					
15–24yr	7,643 (15.3)	293 (11.6)	1,808 (7.8)	270 (5.9)	10,014 (12.5)
25–49yr	26,949 (54.0)	1,258 (49.9)	12,052 (52.2)	2,199 (47.8)	42,458 (53.0)
50–64yr	9,368 (18.8)	474 (18.8)	5,468 (23.7)	1,201 (26.1)	16,511 (20.6)
65–79yr	4,513 (9.0)	379 (15.0)	2,937 (12.7)	540 (11.7)	8,369 (10.4)
80+yr	1,462 (2.9)	117 (4.6)	823 (3.6)	389 (8.5)	2,791 (3.5)
**First dose vaccine**					
Ad26.COV2-S (Janssen)	NA	0 (0.0)	1,134 (5.0)	579 (12.6)	1,713 (5.9)
BNT162b2 (Pfizer/BioNTech)	NA	1,407 (66.8)	14,358 (63.8)	2,932 (64.0)	18,697 (64.0)
ChAdOx1 nCoV–19 (AstraZeneca)	NA	588 (27.9)	5,291 (23.5)	726 (15.8)	6,605 (22.6)
Gam-COVID-Vac	NA	0 (0.0)	86 (0.4)	0 (0.0)	86 (0.3)
mRNA–1273 (Moderna)	NA	111 (5.3)	1,643 (7.3)	347 (7.6)	2,101 (7.2)
Missing	NA	415	576	15	50,941

*Note:* Only cases with a date of onset before week 50 of 2021 were included from Poland and Ireland.

Comparing cases by vaccination status, a higher proportion of partially, fully, or additionally vaccinated cases than non-vaccinated cases were female or older, and a lower proportion were hospitalized (Table 1). Few Alpha, Beta, Gamma, or Delta cases had received an additional dose of vaccination. SARS-CoV-2 variants were reported in distinct waves with Alpha followed by Beta, Gamma, and Delta, which were then followed in turn by Omicron BA.1, and Omicron BA.2 (Figure 1A). Over time an increasing proportion of reported cases were partially, fully, or additionally vaccinated (Figure 1B).

### Adjusted odds ratios between vaccination status and SARS-CoV-2 variants

**Figure 2.**
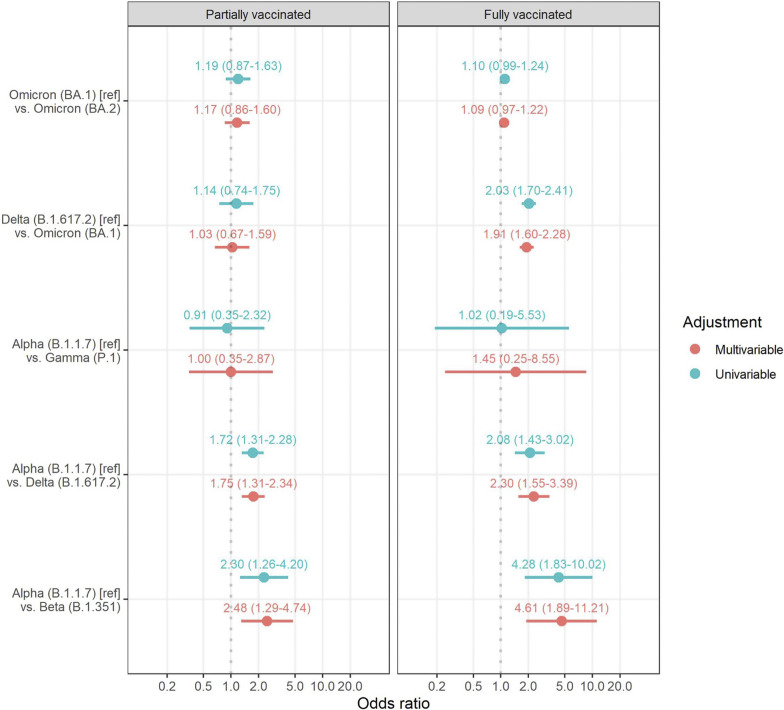
Odds ratios for the SARS-CoV-2 variant comparing partial and full vaccination relative to no vaccination.

Comparing partial vaccination to no vaccination in multivariable conditional logistic regression (see Figure 2), partially vaccinated cases were more likely to be Beta than Alpha, adjusted odds ratio (aOR) 2.48 (95% CI 1.29–4.74; p=0.006), and more likely to be Delta than Alpha, aOR 1.75 (95% CI 1.31–2.34; p<0.001). There was no evidence that partially vaccinated cases were more likely than unvaccinated cases to be Gamma than Alpha (aOR 1.00 95% CI 0.35–2.87; p=0.99), Omicron BA.1 than Delta (aOR 1.03, 95% CI 0.67–1.59; p=0.89), or Omicron BA.2 than Omicron BA.1 (aOR 1.17, 95% CI 0.86–1.60; p=0.33).

For the comparison of full vaccination to no vaccination (see Figure 2), fully vaccinated cases were more likely to be Beta than Alpha (aOR 4.61, 95% CI 1.89–11.21; p<0.001), Delta than Alpha (aOR 2.30, 95% CI 1.55–3.39; p <0.001), and Omicron BA.1 than Delta (aOR 1.91, 95% CI 1.60–2.28; p<0.001). There was no evidence that fully vaccinated cases were more likely to be Gamma than Alpha (aOR 1.45, 95% CI 0.25–8.55, p=0.68), or Omicron BA.2 than Omicron BA.1 (aOR 1.09, 95% CI 0.97–1.22; p=0.15).

For additional dose vaccination, there were only sufficient cases to compare Omicron BA.1 to Delta and Omicron BA.2 to Omicron BA.1. There was evidence that additionally, vaccinated cases were more likely than unvaccinated cases to be Omicron (BA.1) than Delta (aOR 6.16, 95% CI 3.79–10.0, p<0.001). There was no evidence that additionally, vaccinated cases were more likely than unvaccinated cases to be Omicron BA.2 than Omicron BA.1 (aOR 1.05, 0.90–1.24; p=0.52).

Odds ratios from univariable conditional logistic regression, without adjustment for age and sex, were similar to adjusted estimates from multivariable conditional logistic regression (Figure 2).

### Secondary analyses

Comparing different vaccines there was no evidence for a difference in the association between SARS-CoV-2 vaccination status and variant between different vaccines (Appendix Figure 2), but precision was limited. There was similarly no evidence for a difference by period since full vaccination (Appendix Figure 3).

### Sensitivity analysis

Excluding cases that were imported or with missing import status had minimal impact on effect estimates except for the comparison of Omicron (BA.1) to Delta (B.1.617.2), which was reduced toward the null. Confidence intervals were wide reflecting lower precision due to a smaller sample (Appendix Figure 4).

## Discussion

In this analysis of case-only data we find evidence of increased vaccine breakthrough infections with Delta and Beta relative to Alpha from both partial and full vaccination and with Omicron (BA.1) relative to Delta.

Reduced vaccine effectiveness against Beta aligns with findings of 3-fold to 10-fold reduced neutralizing activity of plasma from mRNA-vaccinated individuals and in some cases even greater reductions for ChAdOx1 nCoV-19 (AstraZeneca).[17] In a post hoc analysis of a trial in South Africa, ChAdOx1 nCoV-19 two dose efficacy was estimated at only 10% for symptomatic infection with Beta relative to one dose efficacy of 75% observed before the Beta wave.[18] Lower effectiveness was also observed for Beta relative to Alpha with BNT162b2 (Pfizer/BioNTech) in a Qatari test-negative study.[19] Estimated odds ratios for Delta, Beta, and Omicron (BA.1) were elevated for full vaccination relative to partial vaccination consistent with reduced vaccine effectiveness for these variants following acquired immunity from a second dose.

Lower vaccine effectiveness against Delta than Alpha mirrors findings of reduced neutralization of plasma among individuals vaccinated with BNT162b2 and ChAdOx1 nCoV-19.[20] A test-negative design using UK data reported lower effectiveness against Beta than Alpha for both BNT162b2 and ChAdOx1 nCoV-19.[21] For Omicron, test-negative and cohort designs have indicated lower effectiveness of vaccination relative to Delta for infection and hospitalization.[22–24] We found no evidence for a difference in vaccine breakthrough infections between BA.1 and BA.2 corroborating findings from a UK test-negative study which did not find reduced effectiveness to BA.2.[25]

The correspondence between the results of this study and previous published findings provides further evidence of the value of case-only analysis. Case-only analyses, integrated into routine case-based surveillance can facilitate the rapid and automated assessment of signals of reduced vaccine effectiveness for emerging variants. Unlike test-negative designs, which require information on those testing negative for infection, case-only analyses can be applied with routinely collected case-only surveillance data.

One limitation of this study was the missingness in vaccination status. Given this missing data, we conducted a complete case analysis. Estimates of the variant-vaccination status odds ratio will be unbiased asymptotically under the reasonable assumption that completeness of recording among cases for given covariates does not depend on the variant.[26] The outlined approach can be used for hospitalized cases to assess relative vaccine effectiveness for hospitalization, but in this study, there were too few hospitalized cases to analyze this.

A general limitation of the approach taken is that it provides evidence on the ratio of relative risks between vaccination status and variant, but not on the absolute risk of a vaccine breakthrough infection with a variant. Vaccine effectiveness may be higher for a variant, and yet the risk of infection among the vaccinated is higher, if the risk of infection among the unvaccinated is higher for that variant. A further general limitation is that only variants that circulate concurrently in one or more locations, with a sufficient number of cases for analysis, can be compared.

## Conclusions

Case-only approaches have the potential to provide rapid valuable evidence on relative vaccine effectiveness by variant. Incorporation into routine surveillance would facilitate the detection of signals of reduced vaccine effectiveness for emerging variants. Using a case-only approach applied to European routine surveillance data we found evidence, for increased vaccine breakthrough infections for Delta and Beta relative to Alpha, and Omicron (BA.1) relative to Delta.

## Supporting information

Brown et al. supplementary material Brown et al. supplementary material

## Data Availability

Data from the European Surveillance System (TESSy) will be provided according to data access provisions laid out at https://www.ecdc.europa.eu/en/publications-data/european-surveillance-system-tessy.

## Supplementary material

For supplementary material accompanying this paper visit http://doi.org/10.1017/S0950268824001833. click here to view supplementary material
