# Supplementary material for: Case-only analysis of routine surveillance data: detection of increased vaccine breakthrough infections with SARS-CoV-2 variants in Europe
Source: Epidemiol Infect. 2025 Jan 6;153:e16. doi: 10.1017/S0950268824001833 (PMC11748014; doi:10.1017/S0950268824001833)
Supplement: Brown et al. supplementary material [file S0950268824001833sup001.pdf]

Appendix

Supplementary results

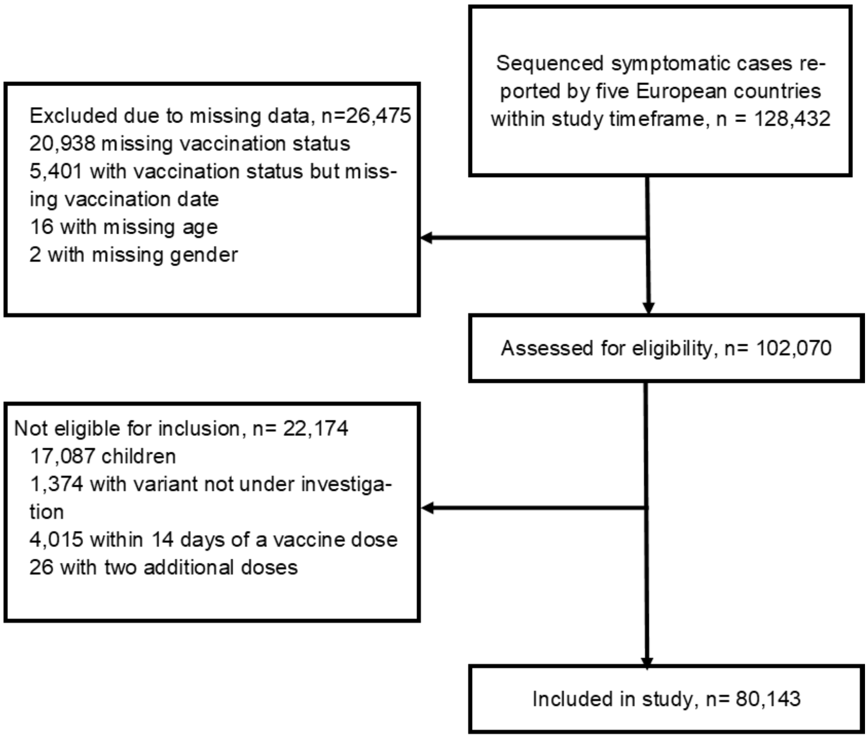

Figure 1: Flow diagram of included cases

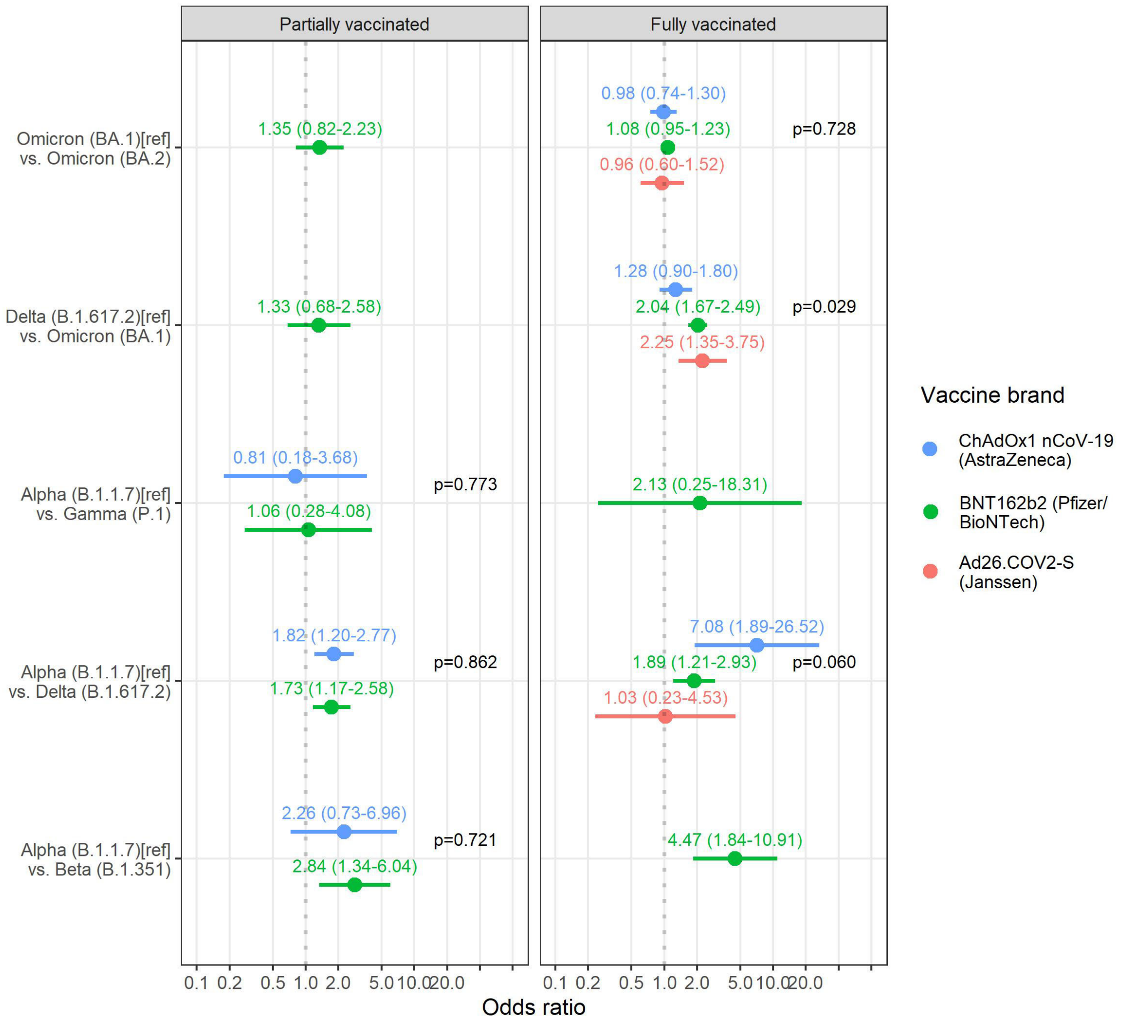

Figure 2: Adjusted odds ratios by vaccine for SARS-CoV-2 variant comparing partial and full vaccination relative to no vaccination

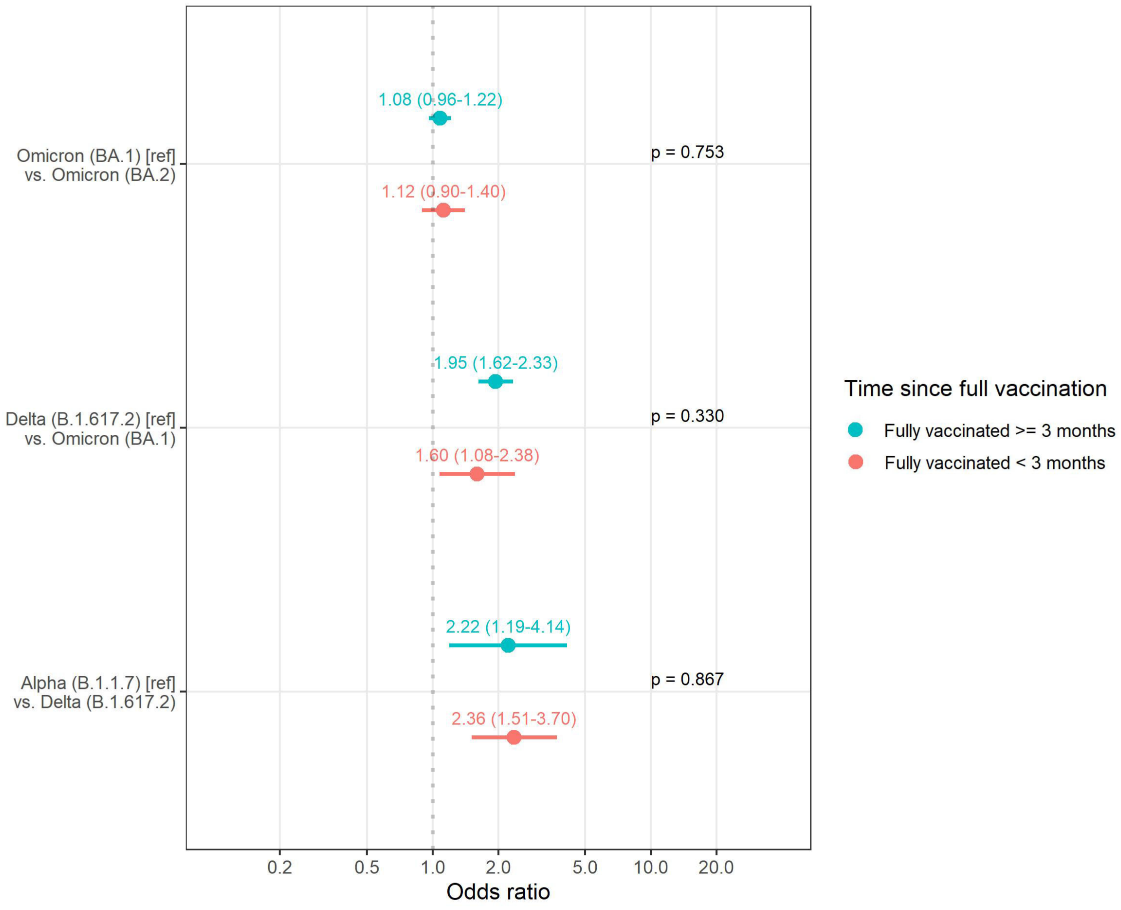

Figure 3: Odds ratios for SARS-CoV-2 variant comparing full vaccination relative to no vaccination by period since full vaccination

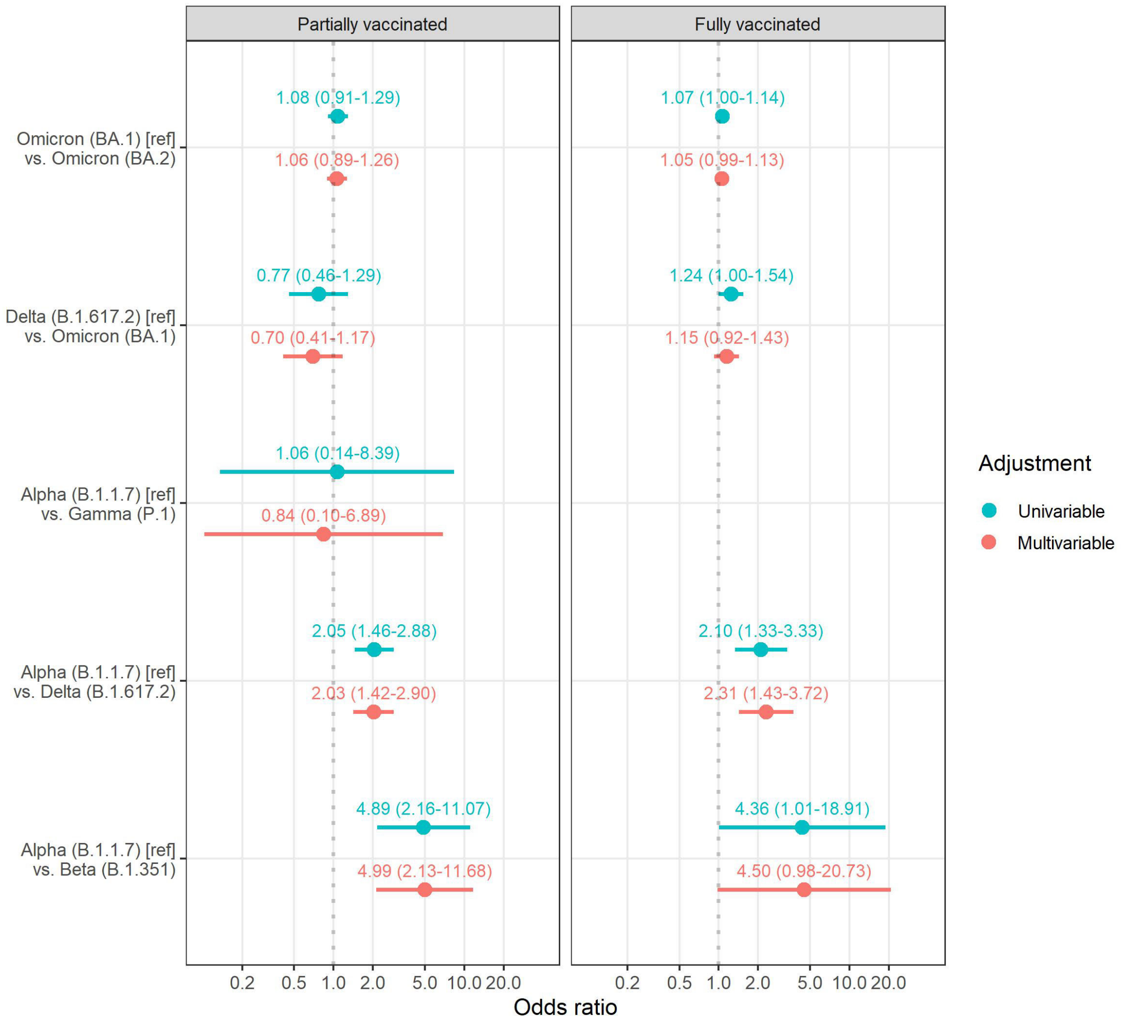

Figure 4: Odds ratios without imported cases for SARS-CoV-2 variant comparing partial and full vaccination relative to no vaccination
